# Hemispheric asymmetries for music and speech: Spectrotemporal modulations and top-down influences

**DOI:** 10.3389/fnins.2022.1075511

**Published:** 2022-12-20

**Authors:** Robert J. Zatorre

**Affiliations:** International Laboratory for Brain, Music, and Sound Research, Montreal Neurological Institute, McGill University, Montreal, QC, Canada

**Keywords:** lateralization, music, speech, neuroimaging, spectrotemporal modulation

## Abstract

Hemispheric asymmetries in auditory cognition have been recognized for a long time, but their neural basis is still debated. Here I focus on specialization for processing of speech and music, the two most important auditory communication systems that humans possess. A great deal of evidence from lesion studies and functional imaging suggests that aspects of music linked to the processing of pitch patterns depend more on right than left auditory networks. A complementary specialization for temporal resolution has been suggested for left auditory networks. These diverse findings can be integrated within the context of the spectrotemporal modulation framework, which has been developed as a way to characterize efficient neuronal encoding of complex sounds. Recent studies show that degradation of spectral modulation impairs melody perception but not speech content, whereas degradation of temporal modulation has the opposite effect. Neural responses in the right and left auditory cortex in those studies are linked to processing of spectral and temporal modulations, respectively. These findings provide a unifying model to understand asymmetries in terms of sensitivity to acoustical features of communication sounds in humans. However, this explanation does not account for evidence that asymmetries can shift as a function of learning, attention, or other top-down factors. Therefore, it seems likely that asymmetries arise both from bottom-up specialization for acoustical modulations and top-down influences coming from hierarchically higher components of the system. Such interactions can be understood in terms of predictive coding mechanisms for perception.

## Introduction

We have known since observations in the mid-19th century about aphasia that the two cerebral hemispheres of the human brain do not have identical functions (Manning and Thomas-Antérion, 2011). Yet, debate continues to this day on the underlying principles that govern these differences. Asymmetries have been described in many domains, including visuospatial, motor, and affective functions. But here I will focus on asymmetries related to auditory processes. A great deal of work has been carried out on the linguistic functions of the left hemisphere, in part because those earliest observations showed such salient effects of left-hemisphere lesions on language in general and speech in particular. But it is instructive to compare speech to that other auditory-motor communication system that we humans possess: music.

Comparisons between music and language are extremely valuable for many reasons (Patel, 2010), and can be carried out at many different levels of analysis. In this mini-review I will focus on certain acoustical features that I argue are critical for important aspects of musical processing, and contrast them with those most relevant for speech, to show that auditory networks within each hemisphere are specialized in terms of sensitivity to those features. However, one of the main points I wish to make is that those input-driven specializations interact with top-down mechanisms to yield a complex interplay between the two hemispheres.

## Specialization for spectral features

A great deal of evidence supports the idea that certain aspects of musical perceptual functions depend to a greater extent on auditory networks in the right hemisphere than the left. This conclusion is supported by a recent meta-analysis of the effects of vascular lesions on musical perceptual skills (Sihvonen et al., 2019), as well as by early experimental studies of the consequences of temporal-lobe excisions (Milner, 1962; Samson and Zatorre, 1988; Zatorre, 1988; Liégeois-Chauvel et al., 1998). Apart from these effects of acquired lesions, deficits in congenital amusia (also termed tone-deafness) also seem to be linked to a disruption in the organization of connections between right auditory cortex and right inferior frontal regions. Evidence for this conclusion comes from studies of functional activation (Albouy et al., 2013) and functional connectivity (Hyde et al., 2011; Albouy et al., 2015), as well as anatomical measures of cortical thickness (Hyde et al., 2007) and of white-matter fiber connections (Loui et al., 2009; Albouy et al., 2013).

These findings are compelling, but what particular aspects of perception are most relevant in eliciting these asymmetries? A hint comes from the amusia literature, where several authors have found that the ability to process fine pitch differences seems to be particularly impaired (Hyde and Peretz, 2004; Tillmann et al., 2016). Those results are echoed in surgical lesion studies showing that damage to an area adjacent to right primary auditory cortex specifically leads to elevated pitch-direction discrimination thresholds compared to equivalent lesions on the left side (Johnsrude et al., 2000). Fine pitch resolution is important for processing musical features such as melody and harmony (Zatorre and Baum, 2012), which is why if that function is impaired, amusia typically follows (Peretz et al., 2002; Hyde and Peretz, 2004).

Many neuroimaging studies also align well with the idea that the right auditory cortical system is specialized for fine pitch processing. Several experiments have found that functional MRI responses in right auditory cortex scale more strongly than those on the left as pitch distance is manipulated from smaller to larger in a tone pattern; that is, the right side is more sensitive to variation of this parameter (Zatorre and Belin, 2001; Jamison et al., 2006; Hyde et al., 2008; Zatorre et al., 2012). Supportive findings also come from an MEG experiment examining spectral and temporal deviant detection (Okamoto and Kakigi, 2015). Importantly, the asymmetry of response seems to be linked to individual differences in pitch perception skill, thus showing a direct brain-behavior link. For example, functional MRI activity in the right (but not the left) auditory cortex of a group of musicians was correlated with their individual pitch discrimination thresholds (Bianchi et al., 2017). A correlation between individual pitch discrimination thresholds and the amplitude of the frequency-following response measured from the right (but not the left) auditory cortex was also observed using MEG (Coffey et al., 2016).

If spectral resolution on the right is better than on the left, what could be the physiological mechanism behind it? One possible answer was provided by an analysis of local functional connectivity patterns in relation to frequency tuning (Cha et al., 2016). This study found that the interconnectivity between voxels in auditory cortex is greater for those whose frequency tuning is more similar than for voxels which are tuned to more distant frequencies. But of greater relevance is that this pattern was more marked within right than left core auditory regions. In other words, frequency selectivity played a greater role on the right than the left, which would then lead to sharper tuning on the right, since there would be summation of activity from neurons with similar response properties. This conclusion is in line with electrophysiological recordings indicating that sharp tuning of neurons to frequency in early auditory cortex depends on excitatory intracortical inputs, rather than thalamic inputs (Liu et al., 2007).

## Specialization for temporal features

The evidence favoring a relative enhancement of frequency resolution in the right auditory networks is paralleled by evidence favoring a relative enhancement of temporal resolution in the left hemisphere. Several functional neuroimaging studies have shown that parametric variation of temporal features of stimuli is better tracked by responses coming from the left auditory cortex and adjacent regions compared to the right (Zatorre and Belin, 2001; Schönwiesner et al., 2005; Jamison et al., 2006; Obleser et al., 2008). Causal evidence in favor of this concept was also provided by a brain stimulation experiment showing increased thresholds for gap detection, after left, but not right auditory cortex disruption (Heimrath et al., 2014).

## Spectrotemporal modulations

A theoretically powerful way to integrate these findings is by considering how these patterns fit with models of spectrotemporal modulation. Many neurophysiological studies exist showing that the response properties of auditory cortical neurons across species are well described in terms of joint sensitivity to spectral and temporal modulations found in the stimulus (Shamma, 2001). This mechanism is thought to enable efficient encoding of complex real-world sounds (Singh and Theunissen, 2003), especially those that are an important part of the animal’s communicative repertoire (Gehr et al., 2000; Woolley et al., 2005). Sensitivity to spectrotemporal modulations in auditory cortex has also been described using both neuroimaging (Schonwiesner and Zatorre, 2009; Santoro et al., 2014; Venezia et al., 2019) and intracortical recordings in humans (Mesgarani et al., 2014; Hullett et al., 2016).

**FIGURE 1:**
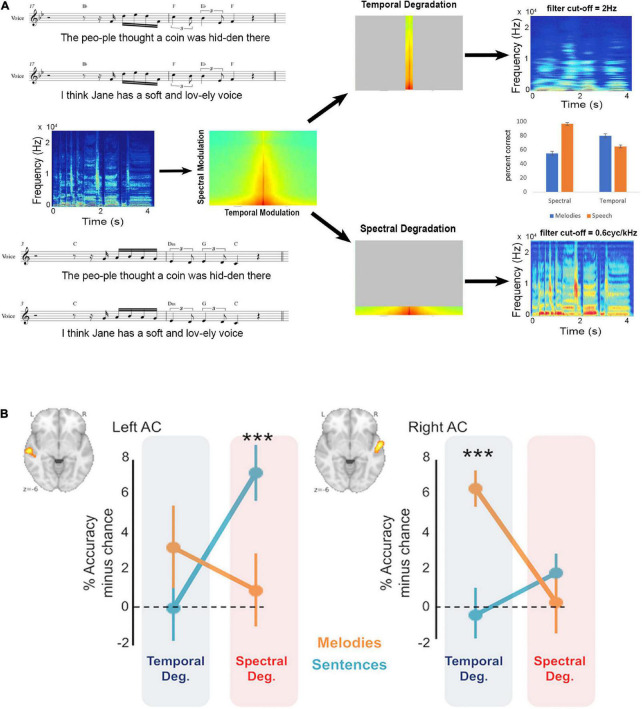
Behavioral and neural effects of spectrotemporal degradation in music and speech. **(A)** Sung stimuli (music notation) consisted of the same tunes sung to different phrases or vice-versa, yielding an orthogonal set of songs with matched melodic and speech content. These songs (spectrogram and spectrotemporal plots in middle panel) were then degraded either in the temporal domain, leaving spectral modulation intact (top), or vice-versa (bottom). The effect of this manipulation can be seen in the resulting spectrograms (right side) where the temporal degradation smears the temporal information but leaves spectral information intact, while spectral degradation smears the spectral information but leaves temporal information intact. The behavioral result (middle panel bar graph) shows that behavioral performance for melodic content is severely reduced after spectral compared to temporal degradation (blue bars) while performance for speech is reduced after temporal compared to spectral degradation (orange bars). **(B)** In the left auditory cortex, functional MRI classification performance for decoding speech content is reduced to chance only after temporal degradation; while in the right auditory cortex functional MRI classification performance for decoding melodic content is reduced to chance only after spectral degradation, paralleling the behavioral effects shown in panel **(A)**. Adapted with permission from Albouy et al. (2020). ***Refers to significantly above chance performance.

Two recent studies have brought together the research questions surrounding hemispheric differences with the spectrotemporal modulation hypothesis to yield evidence that functional asymmetries map well onto this theoretical framework. One study (Flinker et al., 2019) used MEG to measure brain activity associated either with the verbal content, or the timbre (male vs. female voice, which is largely based on spectral cues) of spoken sentences. Behaviorally, they reported that when temporal modulations were filtered out, speech comprehension was affected but vocal timbre was not, and vice-versa for filtering of spectral modulations. The imaging data showed greater left auditory cortex response for the temporal cues in speech, and a right, albeit weaker lateralization effect for the spectral cues. The second study (Albouy et al., 2020) used sung sentences whose speech and melodic content had been fully orthogonalized ensuring independence of the two types of cues (Figure 1). Behavioral data showed a double dissociation such that degradation of temporal cues affected comprehension of the words to the song but not the melody, whereas degradation of spectral cues affected discrimination of the melodies but had no effect on the speech component. The functional imaging data reflected the behavioral data in that speech content could only be decoded from left auditory cortex, but was abolished by temporal degradation, whereas melodic content could only be decoded from right auditory cortex, but was abolished by spectral degradation.

These converging findings from experiments using different techniques strongly support the idea that left and right auditory cortices are linked to heightened resolution in temporal and spectral modulation, respectively. This explanation fits with a broader idea that the nervous system optimizes its representations according to the properties of the physical environment that are most relevant, as has been proposed for vision (Simoncelli and Olshausen, 2001), and for speech (Gervain and Geffen, 2019). I suggest expanding this concept to encompass hemispheric asymmetries on the grounds that humans have two main auditory communication systems, speech and music (Zatorre et al., 2002; Mehr et al., 2021), and that they each exploit, to some extent at least, opposite ends of the temporal-spectral continuum; so the best way to accommodate the competing requirements of the two types of signals is by segregating the necessary specializations within each hemisphere. Thus, rather than think in terms of specializations at the cognitive domain level (speech vs. music), we can reconceptualize it in terms of specialization at the acoustical feature level.

This interpretation predicts that the two domains are lateralized only to the extent that they make greater use of one or another of those cues. We need to keep in mind that both music and speech utilize both temporal and spectral modulations. In the case of speech, spectral modulations are important in carrying prosodic information, and, in tonal languages, lexical information. Interestingly, a good amount of evidence suggests that prosodic processing depends more on right-hemisphere structures, in accord with our model (Sammler et al., 2015). These kinds of spectral cues are important for some aspects of communication, but they do not seem to be quite as important for speech comprehension as those driven by temporal modulations, based on the fact that degradation of temporal but not spectral cues abolishes speech comprehension in the two studies mentioned earlier (Flinker et al., 2019; Albouy et al., 2020). That conclusion was already known from an early influential study (Shannon et al., 1995) that demonstrated that comprehension was well-preserved when normal speech was replaced by amplitude-modulated noise passed through as few as three or four filter banks centered at different frequencies. This procedure degraded the spectral content but preserved most of the temporal modulations. Indeed, this property is what enables cochlear implants to transmit comprehensible speech despite poor representation of spectral modulations due to the limited number of channels available.

Music also contains both spectral and temporal modulations. The latter are obviously critical for transmitting information that can be used to perceive rhythm and metrical organization, and hence the importance of temporal modulations may vary depending on the nature of the music and of the instruments used to generate it (e.g., percussion vs. song). Moreover, pitch information and temporal information interact in interesting, complex ways in music cognition (Jones, 2014). So it is simplistic to think of the two dimensions are entirely independent of one another. However, the fact remains that, as mentioned above, the poor spectral resolution that can be observed with congenital amusia seems to lead to a more global inability to learn the relevant rules of music, and results in a fairly global deficit. So this observation would argue that even if both types of cues are present and important for music, spectral cues seem to play a more prominent role.

## Top-down effects

One might conclude from all the foregoing that hemispheric differences are driven *exclusively* by low-level acoustical features. But that does not seem to be the whole story. There are in fact numerous experiments showing that even when acoustics are held constant, hemispheric responses can be modulated. A good example is provided by studies showing that sine-wave speech analogs elicit left auditory cortex responses only after training that led to them being perceived as speech, and not in the naive state when they were perceived as just weird sounds (Liebenthal et al., 2003; Dehaene-Lambertz et al., 2005; Möttönen et al., 2006). A complementary phenomenon can be seen with speech sounds that when looped repeatedly begin to sound like music (Deutsch et al., 2011; Falk et al., 2014). Once the stimulus was perceived as music, more brain activity was seen in some right-hemisphere regions that were not detected before the perceptual transformation (Tierney et al., 2013). Tracking of pitch contours in speech can also shift from right to bilateral auditory regions as a function of selective attention (Brodbeck and Simon, 2022).

These kinds of results have sometimes been interpreted as evidence in favor of domain-specific models, on the grounds that bottom-up mechanisms cannot explain the results since the inputs are held constant in those studies. However, given the strength of the findings reviewed above that spectrotemporal tuning is asymmetric, another way to interpret these effects is that they represent interactions between feedforward and feedback systems that interconnect auditory areas with higher-order processing regions, especially in the frontal cortex. Although this idea remains to be worked out in any detail, it would be compatible with known control functions of the frontal cortex, which is reciprocally connected with auditory cortical processing streams.

The idea that interactions occur between ascending, stimulus-driven responses, and descending, more cognitive influences can also be thought of in the context of predictive coding models (Friston, 2010). A great deal of work has recently been devoted to this framework, which essentially proposes that perception is enabled by the interface between predictions generated at higher levels of the hierarchy that influence stimulus-driven encoding processes at lower levels of the hierarchy. When the latter signals do not match the prediction, an error signal is generated, which can be used for updating of the internal model (that is, learning). These models have gained prominence because they can explain many phenomena not easily accounted for by more traditional bottom-up driven models of perception, even if they also raise questions that are not yet fully answered (Heilbron and Chait, 2018).

As applied to the question at hand, the idea would be that as a complex stimulus like speech or music is being processed, continuous predictions and confirmations/errors would be generated at different levels of the system. Depending on the spectrotemporal content of the signal, neuronal networks in the left or right auditory cortex would predominate in the initial processing; but as top-down predictions are generated that are based on higher-order features, then the activity could shift from one side to another. So, in the case of sine-wave speech for instance, initial, naïve processing would presumably involve right auditory cortex since the stimulus contains a great deal of spectral modulation. But once the listener is able to apply top-down control to disambiguate how those sounds could fit into a linguistic pattern, then more language-relevant predictions would be generated that could inhibit spectral-based processing in favor of temporal-based processing. By the same token, hemispheric differences could be amplified by these interactions even if initial processing differences in early parts of the auditory system are only slightly asymmetric. This scenario remains largely speculative at the moment, but at least sets up some testable hypotheses for future research.

## Author contributions

RZ wrote the manuscript and approved the submitted version.
